# The Role of Nutrition in Primary and Secondary Prevention of Cardiovascular Damage in Childhood Cancer Survivors

**DOI:** 10.3390/nu14163279

**Published:** 2022-08-11

**Authors:** Fiorentina Guida, Riccardo Masetti, Laura Andreozzi, Daniele Zama, Marianna Fabi, Matteo Meli, Arcangelo Prete, Marcello Lanari

**Affiliations:** 1 Specialty School of Paediatrics, Alma Mater Studiorum, University of Bologna, 40138 Bologna, Italy; 2 Paediatric Oncology and Haematology, IRCCS Azienda Ospedaliero Universitaria di Bologna, 40138 Bologna, Italy; 3 Pediatric Emergency Unit, IRCCS Azienda Ospedaliero Universitaria di Bologna, 40138 Bologna, Italy

**Keywords:** childhood cancer, cancer survivors, antineoplastic-induced cardiotoxicity, anthracyclines, diet-derived compounds, antioxidants

## Abstract

Innovative therapeutic strategies in childhood cancer led to a significant reduction in cancer-related mortality. Cancer survivors are a growing fragile population, at risk of long-term side effects of cancer treatments, thus requiring customized clinical attention. Antineoplastic drugs have a wide toxicity profile that can limit their clinical usage and spoil patients’ life, even years after the end of treatment. The cardiovascular system is a well-known target of antineoplastic treatments, including anthracyclines, chest radiotherapy and new molecules, such as tyrosine kinase inhibitors. We investigated nutritional changes in children with cancer from the diagnosis to the end of treatment and dietary habits in cancer survivors. At diagnosis, children with cancer may present variable degrees of malnutrition, potentially affecting drug tolerability and prognosis. During cancer treatment, the usage of corticosteroids can lead to rapid weight gain, exposing children to overweight and obesity. Moreover, dietary habits and lifestyle often dramatically change in cancer survivors, who acquire sedentary behavior and weak adherence to dietary guidelines. Furthermore, we speculated on the role of nutrition in the primary prevention of cardiac damage, investigating the potential cardioprotective role of diet-derived compounds with antioxidative properties. Finally, we summarized practical advice to improve the dietary habits of cancer survivors and their families.

## 1. Introduction

Despite the significant progress in treatment, in high-income countries, cancer is still one of the major causes of death among children [1,2]. Since 1975, the rate of new diagnosis has been slightly increasing, whereas mortality showed an overall reduction of 68% in children and 59% in adolescents [3,4]. Particularly, the five-year survival rate of acute lymphoblastic leukemia (ALL), the most common childhood malignancy, has increased from under 50% in the 1970s to nearly 85% in the 21st century [5]. The Surveillance, Epidemiology and End Result Program led by National Cancer Institute estimates that the 5-year relative survival of patients who survived cancer is 68.1% from the initial diagnosis [6]. By 2040, the number of cancer survivors is projected to grow to 26.1 million [5]. Indeed, the direct reflection of the reduction in mortality rate is the increasing number of cancer survivors, a fragile population that needs customized clinical attention. The cardiovascular system is one of the most affected targets of antineoplastic toxicity in the short- and the long-term. The incidence of congestive heart failure, myocardial infarction, pericardial disease and valvular abnormalities is significantly increased in patients previously exposed to chemotherapy and thorax-radiotherapy [7]. In the management of cancer survivors, nutrition and dietary habits are well-known key actors in reducing cardiovascular risk, and a customized nutritional intervention to improve dietary habits in children who survived cancer appears to be crucial in secondary cardiovascular prevention [8,9].

Recently, new perspectives have been developing on the role of nutrition in preventing cardiac damage [10,11,12]. An increasing scientific interest is growing in some diet-derived compounds with specific antioxidant properties that can potentially counteract antineoplastic-induced cardiovascular damage during the treatment [13].

In this narrative review, we will provide the reader with an overview on the nutritional interventions in pediatric cancer patients and survivors, with particular interest in the role of nutrition in primary and secondary prevention of cardiovascular damage induced by antineoplastic therapies. We highlighted the burden of acute and chronic cardiotoxicity in this population, dwelling on its potential mechanisms. Moreover, we discussed the alterations of the nutritional status in childhood cancer survivors, suggesting some practical advice for primary and secondary prevention in this fragile population. We also focused on the available evidence about the potential protective role of several diet-derived compounds against chemotherapy-related cardiotoxicity.

## 2. Cardiovascular Damage in Cancer Patients: Acute and Chronic Cardiotoxicity

Advances in cancer therapy pursue the aim of maximizing the cure while reducing the risk of late effects [6]. Indeed, the cumulative incidence of cardiac death 15 years after cancer diagnosis is decreasing from 0.5% for those diagnosed in the 1970s to 0.1% for those diagnosed in the 1990s, as assessed by the North American Childhood Cancer Survivor Study covering over 30,000 long-term survivors of childhood cancer [14]. Nevertheless, the cumulative incidence of chronic cardiovascular disease 15 years after primary cancer diagnosis among 5-year survivors remains relevant [15] and did not significantly decrease over years for heart failure, myocardial infarction and thromboembolic disease [16]. Particularly, 30 years after anthracyclines and radiotherapy exposure, symptomatic cardiac events as congestive heart failure, cardiac ischemia, valvular disease, arrythmia and pericarditis affects one in eight cancer survivors [17].

To estimate the incidence of cardiovascular events according to different types of chemotherapy is even more difficult, varying from >20% of patients treated with anthracyclines to <5% of children receiving alkylating agents such as Cisplatin [18].

Based on time of presentation, anthracycline-related cardiotoxicity (ACT) can be categorized as acute, early and late-onset. Acute toxicity occurs in less than 1% of children within a few hours from drug infusion and its manifestations, including arrhythmias and a myocarditis-pericarditis syndrome, and are usually reversible. Early-onset toxicity usually occurs within the first year of treatment as a dilated cardiomyopathy with decreased left ventricular wall thickness and global contractility [19]. The progression of this cardiac alteration or its onset one year after the end of anthracycline therapy defines the late-onset toxicity [20]. In this case, a progressive pathologic cardiac remodeling occurs, resulting in a reduction of left ventricular dimensions and an increase of the posterior wall thickness, also described as “Grinch syndrome”. The right ventricle is less commonly affected [19].

Similarly, the cardiotoxicity related to other conventional cytotoxic chemotherapy or to newer molecularly targeted agents can be categorized in acute and early-onset if it occurs within the first year of cancer therapy or chronic cardiotoxicity if it occurs later [15].

Depending on the specific agent considered, acute toxicity described in children includes arrhythmia, transient or progressive left ventricular systolic dysfunction, myocarditis, pericarditis and vascular complications, such as hypertension and ischemia. Subclinical troponin elevation related to myocardial injury is amongst the most common effects, being observed in almost 50% of children treated with moderate dose anthracyclines for ALL [21]. In children diagnosed with acute myeloid leukemia (AML) and treated with high doses anthracyclines, left ventricular systolic dysfunction occurred in the 12% of patient with more than 70% of cases developed during the treatment [21]. The incidence of myocarditis has been estimated to be 5% of children treated with high dose cyclophosphamide [18,22]. Tyrosine kinase inhibitors may determine acute hypertension and QTc prolongation in 2–10% of children treated [15].

The prevalence of chronic cardiovascular damage is difficult to determine and may differ according to the clinical entity considered. Symptomatic cardiac dysfunction has been estimated to be around 16% among anthracyclines-exposed survivors, even though subclinical disease can occur in over 50% of children [18]. Moreover, chronic cardiomyopathy is combined with other cardiovascular alterations in 25% of childhood cancer survivors and it is associated with two or more modifiable cardiovascular risk factors in up to 10% of cases [16].

Cardiac events were found to be significantly more frequent in young survivors of cancer than in siblings and the prevalence of congestive heart failure was reported to be almost eights time higher at a mean age of 27 years (1.7% in cancer survivors versus 0.2% in siblings) [23]. Indeed, the risk of developing congestive heart failure is 4.9 times higher in children previously treated for cancer than their siblings [24]. The risk of developing valvular abnormalities and pericardial disease is respectively three times and four times higher in children treated rather than their siblings [24]. Children that develop heart failure during and/or after cancer treatment have an almost four times greater mortality risk than for children with cancer alone and the difference is even more significant when pediatric patients are compared to adults [23]. Regarding this comparison, it is difficult to assess whether children are at higher mortality risk for cardiovascular effects related to cancer treatment rather than adults. In childhood, additional comorbidities or competing causes of death are less frequent than in adults, making more dramatic the relative effect of developing heart failure. Furthermore, children are less likely to tolerate or respond to heart failure treatment and their heart could be more sensitive to the effects of cancer treatment [23].

Among the 13,060 participants in the Childhood Cancer Survivor Study, ischemic heart disease and stroke occurred in 265 and 295 childhood cancer survivors through age 50 years, respectively [25]. Radiation to the heart and exposure to anthracyclines were risk factors for ischemic heart disease, heart failure and stroke. Radiation to the heart, the brain and neck was a risk factor for stroke [25]. Furthermore, alkylating and similar DNA interstrand cross-linking such as cisplatin have been associated with an increased risk of stroke as a potential result of release of prothrombotic complexes after administration [25].

Arterial hypertension is amongst the most common cardiovascular toxicity in cancer survivors, reported with a prevalence of 37% [26], especially for newly developed target therapy against vascular endothelial growth factor (i.e., Bevacizumab), platelet-derived growth factor (i.e., Sunitinib) and fibroblast growth factor apparently in a dose-dependent manner [27].

Pericardial disease and particularly pericardial effusion and/or constriction occur in 6–30% of patients after radiation therapy. Acute pericarditis usually occurs as an acute complication of chest irradiation (within months) and it is often self-limiting whereas chronic pericarditis manifests as an effusive-constrictive disease [28].

Lastly, hematopoietic stem cell transplantation is also burdened by significant cardiotoxicity, probably as a consequence of high-doses antineoplastic treatment combined with irradiation [29]. In these patients, the cumulative incidence of coronary artery disease, cerebrovascular accident, cardiomyopathy and cardiac-related death was found to be equal to 0.2%, 0.6%, 3% and 0.5%, respectively [30].

## 3. Mechanisms of Antineoplastic-Induced Cardiotoxicity

**Figure 1:**
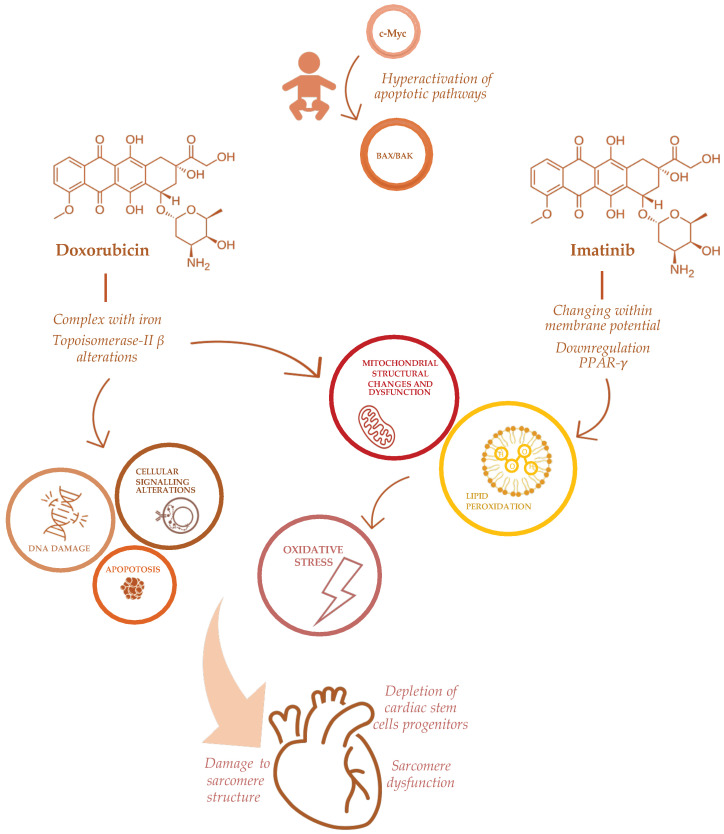
Mechanisms of doxorubicin and imatinib-induced cardiotoxicity in childhood cancer patients.

The complex pathogenesis of antineoplastic-induced cardiac damage has been deeply investigated [31,32,33]. Cardiomyocytes have a limited regenerative ability that typically expose them to persistent long-term damage due to antineoplastic drugs. In the pediatric population, the effect of cardiac damage is even more complicated, varying from different drug metabolisms to intracellular diversity [28]. Unlike adults, children’s cardiomyocytes express an apoptotic pathway involving *c-Myc* via *BAX/BAK* effectors that could explain their hypersensitivity to antineoplastic drugs, specifically doxorubicin [34] (Figure 1). Indeed, a sophisticated mechanism has been described in mice, involving the mitochondrial response to genotoxic agents [34].

Among chemotherapeutic agents, anthracyclines such as doxorubicin, epirubicin, and daunorubicin remain drugs of choice for many cancer treatments, and cardiotoxicity is a well-known adverse effect of these medications [7].

ACT has been directly related to anthracyclines’ mechanism of action, involving the formation of complexes with iron, impairment of oxidation and reduction processes with subsequent increasing production of free radicals (ROS) [7]. Other effects of anthracyclines on cardiomyocytes include significant depletion of cardiac stem cells progenitors [35], a profound impairment in apoptosis and cell signaling that triggers cell death [36] and DNA synthesis [37], damaging sarcomeres’ function [38] and mitochondrial activity [7], also via Topoisomerase-II β alterations [19]. Furthermore, anthracyclines determine a direct toxicity on the sarcomeres’ structure, inhibiting calcium release from the sarcoplasmic reticulum [37], impairing formation of protein titin and the activity of mitochondrial creatine kinase [39] (Figure 1).

ACT severity can differ among patients, suggesting a different susceptibility to the cardiotoxic effects of anthracyclines. Genetic predisposition has been investigated in the pathogenesis of ACT, advocating the possible use of pharmacogenomic testing before the start of treatment [7]. In this perspective, an ACT risk prediction model, based on genetic and clinical information, has been developed [40].

Indeed, well-established risk factors for ACT are: female sex, African ancestry, trisomy 21, young age at cancer diagnosis (especially <4 years of age), chest radiation and cardiovascular risk factors [7]. Furthermore, the risk of ACT can be increased by different therapeutic strategies, such as higher anthracyclines cumulative dose (greater than 500 mg/m^2^), intravenous bolus injection rather than continuous infusion therapy and the use of liposomal vectors [41].

Although ACT is probably the best known antineoplastic-induced cardiotoxicity, other chemotherapeutic agents are potentially responsible for cardiovascular damage, including anti-metabolites and 5-fluorouracil, which can induce myocardial ischemia via coronary vasospasm and pericarditis [19]. Alkylating agents can lead to endomyocardial fibrosis, pericarditis, hypertension and left ventricular dysfunction, worsening ACT when combined with anthracyclines [19].

Furthermore, several emerging antineoplastic therapies, recently approved in the pediatric population, may be responsible for cardiac adverse effects. Tyrosine kinase inhibitors are novel small molecules that interfere with molecular pathways involved in cellular proliferation, differentiation and survival. Their adverse events on the cardiovascular system, hypertension, thromboembolism, pulmonary hypertension and ventricular dysfunction are described [42]. Specifically, imatinib, a first-generation tyrosine kinase inhibitor, approved for chronic myeloid leukemia, induces cardiotoxicity altering mitochondrial function through changes within the membrane potential, impairing endoplasmic reticulum response to stress, promoting apoptotic pathways and increasing reactive oxidative species production [43]. The specific mechanism of imatinib-induced cardiotoxicity is through the down-regulation of peroxisome proliferator-activated receptor-γ (PPAR-γ) levels, with a subsequent alteration in carnitine homeostasis, mitochondrial dysfunction and decreased ATP generation [43]. Through the same mechanism, imatinib seems to increase oxidative stress and the production of nitric oxidative species in vessels, thus leading to endothelial dysfunction in vivo [44] (Figure 1).

Sorafenib, an inhibitor targeting FLT3 approved for children with AML, may determine significant hypertension and left ventricular systolic dysfunction, particularly when combined with anthracyclines, due to its off-target activity on the vascular endothelial growth factor [45].

Additionally, other antineoplastic medications, such as immune checkpoint inhibitors, have been associated with rare but fatal myocarditis and arrhythmias. Finally, the novel chimeric antigen receptor T-cells therapy could induce a massive cytokine release syndrome with subsequent vasoplegic shock and ventricular dysfunction [42].

## 4. Nutritional Status Impairment in Pediatric Cancer Patients

The profound alterations of the nutrition status frequently observed in pediatric cancer patients during and after chemotherapies have a potentially great impact on the future cardiovascular risk of these patients [10].

Malnutrition and significant weight loss have been commonly observed during cancer therapy, especially in children with ALL/lymphoblastic lymphoma and AML [46,47]. Particularly, malnutrition in cancer patients is determined by a combination of reduced food intake and metabolic alterations such as elevated resting metabolic rate, increased catabolic factors and systemic inflammation [48]. The subsequent negative energy balance and skeletal muscle loss with increased lipolysis and proteolysis aggravate weight loss and create a vicious cycle that can only be partially reversed by conventional nutrition support [48]. Malnutrition in childhood cancer patients is also associated with poorer prognosis [10]. Indeed, significant weight loss during induction therapy has been associated with gastrointestinal and hepatic toxicity and a greater risk of major infection [37].

Nevertheless, the same population experiences overweight and obesity during survivorship, without referring to a dietician during treatment or in the next 3 years of follow-up [46]. Some authors reported that children diagnosed with ALL are at higher risk of rapid gain weight during induction and during the first 6 six months of maintenance therapy, persisting beyond the end of treatment [49].

Several studies investigated the nutritional status of cancer survivors focusing on dietary habits and the reasons behind the risk of overweight [9,46,47,50,51,52,53] that expose these children to cardiovascular diseases and related mortality seven times more than the general population [49]. Furthermore, overweight and obesity could significantly affect anti-neoplastic drugs’ pharmacokinetic properties, increasing the risk of cardiotoxicity, as higher body surface area has been associated with increased risk of ACT [47].

There are several mechanisms underlying the risk of overweight in cancer survivors.

Firstly, corticosteroids are frequently included in several antineoplastic regimens. A prolonged use of these drugs is a common cause of increased adipose tissue deposition with a catabolic effect on muscular tissue. Corticosteroids are also responsible for endocrine disturbance, appetite fluctuation and sudden mood fluctuations that frequently lead to inappropriate food consumption. Together with L-asparaginase, a cytoreductive agent included in pediatric ALL and lymphoma protocols, corticosteroids may also impact lipid metabolism, particularly causing hypertriglyceridemia [54]. Moreover, children with ALL and brain tumors are at risk for hypothalamic-pituitary axis damage secondary to cranial irradiation, brain surgery or to primary tumor location with consequently higher risks for obesity and metabolic syndrome [55].

Anthracyclines can cause left ventricular dysfunction with subsequent impairment in cardiovascular fitness. Other neurotoxic agents commonly co-administered with anthracyclines, such as vincristine, can exert peripheral neuropathy, thus worsening muscle strength and exacerbating mobility limitations [49].

Furthermore, cancer survivors are usually sedentary with poor levels of physical activity and a large positive energy gap (approximately 500 kcal/day lower than the estimated energy requirement). Indeed, childhood cancer survivors present poor diet quality and weak adherence to dietary guidelines [49]. Particularly, a higher daily amount of sodium and fat and a lower consumption of fish, fruit and vegetables with a low level of potassium intake were reported among cancer survivors [9,51]. As for micronutrients, total vitamin D intake appears to be below the recommended value in this population, even though these patients are not more prone to vitamin D insufficiency or deficiency than the general population [56]. Regarding lipid quality, consumption of polyunsaturated and monounsaturated fats are lower than the general population, whereas the level of saturated fats assumption is close to the upper limit [50].

In addition, permissive parenting behavior is common nowadays, leading to unhealthy dietary habits and sedentary lifestyles [49].

Hypertension, dyslipidemia, diabetes and obesity, combined or alone, have been identified as modifiable cardiovascular risk factors for childhood cancer patients and their early detection, and along with a prompt treatment, can substantially reduce the risk of premature cardiac disease [57].

## 5. Primary Prevention of Cardiovascular Damage in Childhood Cancer Survivors: Is There a Role for Nutrition?

### 5.1. Potential Role of Diet-Derived Compounds in Primary Prevention of Cardiac Damage: What Is New

Amongst the multifaceted connection between nutrition and cancer, could nutrition and dietary intervention be effective in reducing cardiotoxicity itself?

So far, just a few molecules demonstrated their efficacy in the primary cardioprotection of children treated with anthracycline-containing chemotherapy, and dexrazoxane is the most well-studied amongst them. It is a synthetic iron-chelating agent that reduces the formation of iron-anthracycline complexes [58], and whose clinical use as a cardioprotective agent has been approved for adults and widely investigated in children [59,60,61]. In patients under the age of 18 years, dexrazoxane is not licensed, but recently, the European Medicines Agency removed this contraindication for childhood cancer patients treated with high cumulative doses of anthracyclines (more than 300 mg of doxorubicin per m^2^ body surface) [62]. Indeed, a recent report from the Children’s Oncology Group demonstrated that dexrazoxane prevented cardiac alteration in children treated for AML without increasing relapse risk or second cancers [63] and the same brilliant effect has been proven in children with ALL, osteosarcoma and Hodgkin’s lymphoma [64].

A group of diet-derived compounds seems to exhibit a cardioprotective role in chemotherapy-related toxicity due to their antioxidant, free radical scavengers and metabolic properties.

However, most of the studies conducted so far are preclinical in vivo and in vitro research, without enough strength to recommend these molecules in clinical practice.

Here, we report a list of the most widely studied compounds (see Table 1) that can exert a cardioprotective effect and that can be potentially useful in the primary prevention of chemotherapy-related cardiotoxicity. The effect of ω-3 polyunsaturated fatty acids (PUFA) is suited for this purpose. As well as PUFAs, other dietary products, such as ubiquinone, flavonoids and polyphenols, some vitamins, amino acids and micronutrients proved to have cardioprotective properties, and they are currently under investigation for potential nutritional supplementation during chemotherapy.

#### 5.1.1. Polyunsaturated Fatty Acids (PUFA)

These molecules cannot be produced by the human body and their levels depend on dietary intake, especially on consumption of marine fish and vegetable oils and seeds (such as flax, canola and soy).

ω-3 PUFAs are membrane phospholipids and play an important role in intracellular and cell-to-cell signaling, modulating gene expression. Furthermore, they actively participate in eicosanoid metabolism, contributing to cell growth and differentiation, immunity, inflammation, platelet aggregation and angiogenesis. They seem to have antioxidantidant and anti-inflammatory properties, directly inhibiting carcinogenesis and tumor expansion through their anti-angiogenetic effects [54]. ω-3 PUFA are also involved in maintaining gut health, preserving enterocytes’ structure and intestinal mucosal barrier. In adults, ω-3 PUFA demonstrate an improved tolerance to common antineoplastic adverse effects as nausea, appetite loss and fatigue [54].

Despite their anti-inflammatory and antioxidant effects, these molecules could play a role in preventing lipid dysregulation, alteration in glucose metabolism, and low-grade inflammation related to the long-term disease following cytotoxic therapies [54], and even ACT.

The administration of ω-3 PUFA seemed to prevent lipid peroxidation and reduce oxidative stress in animal models with acute cardiotoxicity induced by doxorubicin [98].

Conversely, other in vivo studies showed that fish oil dietary supplementation in rats administered with doxorubicin was associated with higher mortality rates and a lower cardiac performance with respect to rats treated with doxorubicin alone. Indeed, in these models, dietary fish oil seemed to enhance lipid peroxidation and reduce antioxidant defenses through decreasing myocardial vitamin E levels [69]. A similar unfavorable effect was described in merino sheep treated with doxorubicin, in which the coadministration of ω-3 PUFA worsened left ventricular dilatation and systolic function [69]. However, a recent study conducted in children diagnosed with ALL, an oral supplementation of ω-3 PUFA (1000 mg/day for 6 months) started one week before the beginning of chemotherapy, significantly decreased early doxorubicin-induced cardiotoxicity. Particularly, children that received ω-3 PUFA as a pre-treatment and during chemotherapy did not show echocardiographic signs of left ventricular systolic dysfunction (defined as alterations in two-dimensional global longitudinal strain and in left ventricular peak mitral annulus systolic velocity) neither subclinical cardiac damage (defined as increased Troponin-I, creatine kinase-MB and *N*-terminal pro-brain natriuretic peptide serum levels). Furthermore, oral administration of ω-3 PUFA reduced the evidence of doxorubicin-induced oxidative stress, increasing serum levels of superoxide dismutase and glutathione and reducing the level of malondialdehyde [63].

#### 5.1.2. Ubiquinone

Ubiquinone or coenzyme Q_10_ (CoQ_10_) is a hydrophilic molecular component of the electron transport system, mainly located into the inner mitochondrial membrane. CoQ_10_ is synthesized by mammals from tyrosine metabolism, and it is implementable through alimentary consumption of oily fishes and whole grains. Intracellular amount of CoQ_10_ reflects the cellular content of mitochondria. In cardiomyocytes, CoQ_10_ reaches high concentration, approximately 5 times higher than that in the liver and 10 times that in the kidneys, pancreas and spleen. Furthermore, CoQ_10_ is located inside the plasma membrane and in many other intra-cytoplasmic membranes including that of the Golgi apparatus, the endoplasmic reticulum and lysosomes. As a substantial intramembrane component, it acts as a peroxyl radical scavenger, inhibiting lipid peroxidation and protecting mitochondrial proteins against oxidative stress. CoQ_10_ also has an antioxidant effect on low-density circulatory lipoproteins, preventing lipid peroxidation, which is an inconvenient effect of a diet rich in polyunsaturated fatty acid [11]. Interestingly, CoQ_10_ exhibits a cytostatic effect against hepatocellular carcinoma induced by toxic agents in animal models. Lower plasmatic level of CoQ_10_ has been observed in patients with breast and myeloma cancer [64]. For its properties, a few clinical and preclinical studies showed the protective effects of CoQ_10_ against the chronic cardiotoxicity induced by doxorubicin, without any interference with anthracyclines’ antitumor activity [13,41]. Particularly, in cultured mouse myocardial cells, doxorubicin increased to 50% levels of products of lipid peroxidation such as malondialdehyde. In the same in vitro model, the concomitant addition of CoQ_10_ maintained levels of malondialdehyde equal to that of the control culture, without reducing the uptake of doxorubicin by myocardial cells [99]. Furthermore, the administration of ubiquinone in children diagnosed with ALL or non-Hodgkin lymphoma and treated with anthracyclines seemed to reduce ACT through lightening the alteration of septum wall mobility and the reduction of left ventricular ejection fraction mediated by doxorubicin [67].

#### 5.1.3. Flavonoids and Polyphenols

Flavonoids as catechins, quercetin and genistein, as well as polyphenols such as curcumin and resveratrol, which have been proven to have similar anti-oxidative properties.

Flavonoids are naturally occurring in green tea, some fruits (berries, apples, pears, peaches and avocados) and some nuts (pecans, pistachios and hazelnuts). They act as iron chelators and as reactive oxygen species scavengers, in a dose-dependent mechanism [13]. Through these properties, these molecules have been studied in the prevention of ACT in preclinical studies.

Epigallocatechin gallate (EGCG), a catechin highly concentrated in green tea, exhibited antimutagenic, antiangiogenic, antiproliferative and proapoptotic activity in mammalian cells, in both in vivo and in vitro studies [100]. Particularly, it seems that EGCG directly counteracts reactive oxygen species formation induced by doxorubicin in a concentration-dependent manner in in vitro models [101]. Indeed, EGCG significantly reduces reactive oxygen and nitrogen species and intracellular damage through increasing the activity of cytochrome P-450 reductase in the liver, heart and lungs [13,68]. Furthermore, a pretreatment with EGCG seemed to decrease iron accumulation and ferroptosis induced by doxorubicin in in vitro models [69]. Lastly, EGCG seems to exhibit a synergistic anti-cancer effect when combined to antineoplastic drugs through a complex mechanism that comprehends cancer stem cells inhibition, apoptosis regulation and genetic and epigenetic modulation [100]. Indeed, in in vitro and in vivo models, EGCG significantly increased intracellular apoptosis of cancer cells and inhibited tumor growth when combined with paclitaxel [101].

Quercetin, a pigment that gives to some vegetables and fruits a distinctive color (i.e., citric fruits, tea, olive oil and berries), similar to other flavonoids, has antioxidant properties. It also inhibits topoisomerase II-β, intercalates itself into DNA strands and increases the concentration of doxorubicin inside cancer cells. In many studies, quercetin showed antitumor properties through direct and indirect effects, including clastogenic effects and the decrease of the resistance to chemotherapy drugs [70,71,72,102,103]. Furthermore, cytoprotective effects were shown in the heart, liver and spleen [73]. Specifically, quercetin was found to strongly inhibit the formation of doxorubicinol, the main metabolite considered to be responsible for chronic cardiotoxicity of doxorubicin, suggesting a deep cardioprotective effect in patients treated with anthracyclines [74].

Similar properties have been exhibited by genistein, a soy-derived isoflavone, that specifically inhibits topoisomerase II-β and enhances doxorubicin intracellular accumulation, showing antioxidant properties [13]. Particularly, genistein seems to counteract doxorubicin-induced apoptosis and inflammation, reducing level of *TNF-α, IL-6* and *IL-8*, downregulating the expression of survival proteins (*p-Akt, Bcl-2*) and upregulating proapoptotic pathways (*Erk, Bax, cleaved caspase-3*) [75]. Indeed, in animal models, oral administration of genistein along with intraperitoneal doses of doxorubicin significantly improved cardiac function markers and reduced oxidative stress markers [75].

Curcumin, a phenolic pigment responsible for the yellow color of the curry spice turmeric, inhibits lipid peroxidation through its free radical scavengers’ role, increases intracellular level of glutathione, contributes to cholesterol homeostasis, and stabilizes cardiac cell membrane [104]. In animal models, the oral supplementation of curcumin proved to reduce doxorubicin toxicity in the heart, livers and kidneys [13]. In addition, in animal models, the oral administration of curcumin before intraperitoneal doxorubicin injection reduced cardiac alteration, prevented the elevation of cardiac enzymes [77] and decreased oxidative stress [78] induced by anthracyclines alone. Interestingly, curcumin exhibits a cardioprotective role in other cardiovascular diseases as demonstrated in animal models of myocardial ischemia [79] and diabetes in which it counteracted oxidative stress [79] and endothelial alterations [80,81].

Resveratrol, a non-flavonoid polyphenolic compound, naturally present in grapes, red wine, chocolate, peanuts and some berries, demonstrated in vitro antioxidant, anti-inflammatory and anticancer effects, targeting well-known tumor suppressors (*p53* and *Rb*) and cell cycle mediators [13,105]. Particularly, the coadministration of resveratrol and doxorubicin in mice models prevented the elevation of blood pressure and compensated cardiac hypertrophy induced by anthracyclines alone through anti-inflammatory effects [82]. In in vivo studies, pretreatment with resveratrol protected cardiac cells from oxidative and electrophilic cell injury induced by doxorubicin [83] and improved cardiac function, restoring a normal heart rate, left ventricular ejection fraction and fractional shortening [84]. The same cardioprotective effect was also demonstrated in an in vitro study in which resveratrol reduced the expression of *E2F1/mTORC1* and *E2F1/AMPKα2* pathways, thus inhibiting cardiomyocytes apoptosis induced by doxorubicin [85]. Furthermore, resveratrol seems to counteract chronical cardiac damage induced by doxorubicin, restoring the ability of the hearth to develop a compensatory cardiac hypertrophy in response to hypertension in mice injected with anthracyclines that had lost this adaptive mechanism [86]. However, resveratrol properties have not been fully confirmed in humans [106].

#### 5.1.4. L-Carnitine and Glutathione

L-carnitine, an amino acid found in both vegetal and animal food sources, plays a substantial role in the transportation of long-chain fatty acids across the mitochondrial membrane, and is also involved in lipid and amino-acid metabolism [43]. It reduces lipid peroxidation of cardiac membranes and reduces anthracyclines’ ability to inhibit long-chain fatty acids production [41]. In animal models, L-carnitine seems to counter imatinib-induced cardiotoxicity, scavenging free radicals, reducing apoptotic activation and decreasing levels of pro-inflammatory markers, not only in cardiomyocytes but also in endothelial cells [43]. Furthermore, L-carnitine seemed to reduce the expression of oxidative reactive species in mice treated with cisplatin [87] and to reduce echocardiographic changes and histopathological alterations induced by doxorubicin in rats [88].

Glutathione, a tripeptide whose levels may be influenced by dietary and supplemental nutrients [107], acts as a free radical scavenger that directly protects cardiac cells against oxidative stress [12,41]. The cardioprotective role of glutathione has been investigated in the past twenty years as the administration of anthracyclines is associated with drug-related depression in glutathione peroxidase activity in cardiac cells and a subsequent reduction of glutathione levels [108]. In animal models, glutathione reduced lipoprotein oxidation and histopathological alterations in mice injected with doxorubicin, counteracting the elevation in heart rate, blood pressure and cardiac markers induced by anthracyclines alone [89].

#### 5.1.5. Vitamins

Lastly, also liposoluble (vitamin A, vitamin E) and hydrosoluble (vitamin C) vitamins have been studied in the primary prevention of cardiotoxicity due to their antioxidant properties.

Preformed vitamin A (retinol, retinyl esters) and provitamin A carotenoids (i.e., beta-carotene converted to retinol) are highly concentrated in multiple cereals, orange and yellow vegetables, many fruits, and in olive and fish oils.

In in vivo studies, a pretreatment with all-trans-retinoic acid which is a natural derivative of vitamin A, prevented cardiac biomarker elevation, reduced the expression of pro-apoptotic pathways (*caspase 3, p53*) and reduced proinflammatory cytokines and lipid peroxidation induced by doxorubicin [90].

In other animal models, vitamin A shows a protective dose-dependent effect against chromosomal aberration induced by anthracyclines [13,44].

Lycopene is a carotenoid with a powerful antioxidant activity. In animal models, the contemporary intraperitoneal administration of lycopene and anthracyclines seems to decrease malondialdehyde and reduces glutathione levels in both the heart and kidneys, preventing cardiac and renal histopathological alterations observed in animals treated with anthracyclines alone [91].

Furthermore, intraperitoneal administration of lycopene in animal models of acute doxorubicin myocardial toxicity prevented the elevation in cardiac markers and slighted myocardial inflammation induced by doxorubicin alone [92]. Interestingly, lycopene also exhibits protective properties in testicular toxicity and the consequential sterility induced by anthracyclines in animal models [93].

Vitamin E, and particularly α-tocopherol, is found in plant-based oils, seeds and many fruits and vegetables. Its antioxidant effects are well-known in many biological processes. The role of vitamin E in the potential prevention of cardiotoxicity is controversial. Pre-treatment with vitamin E in in vitro models of *Nox-1* overexpressing prostate tumor cells incubated with vitamin E increased intracellular level of P-glycoprotein and of hypoxia inducible factor-1 α, reducing the antitumor activity of anthracyclines [94]. High doses of vitamin E contrasts lipid peroxidation and chromosomal aberrations without preventing chronic ACT, and does not significantly reduce acute cardiotoxicity [11]. Moreover, in rats, the administration of vitamin E before the injection of doxorubicin seems to prevent electrocardiographic changes induced by anthracyclines, lowering creatine phosphokinase and lactate dehydrogenase levels that were previously increased by anthracyclines [95].

Vitamin C or ascorbic acid is typically found in citrus fruits, even though it is not constricted to just them. It may be also found in many other fruits and vegetables, such as strawberries, cruciferous vegetables, bell peppers, white potatoes and tomatoes. Similar to other vitamins, it has antioxidant properties and contributes to vitamin E regeneration, thus potentiating its effects [12]. In in vivo studies, administration of vitamin C seems to improve survival of mice injected with doxorubicin improving cardiac function and histological damages via its antioxidative, anti-apoptotic and anti-inflammatory effects [96]. Nevertheless, the role of vitamin C as a cardioprotective agent, alone or combined with vitamin E, has not been fully demonstrated in humans [97].

### 5.2. Nutritional Recommendations during Cancer Treatment in Children

Although many studies have been conducted on the potential role of new dietary compounds in reducing the risk of drug-induced cardiotoxicity, evidence is still too weak to recommend the regular consumption of these dietary supplements during cancer treatment as cardioprotective agents.

Nevertheless, general nutritional recommendations during cancer treatment in children have been developed. In 2016, the European Society for Clinical Nutrition and Metabolism (ESPEN), along with the European Partnership for Action Against Cancer (EPAAC), produced guidelines for the nutritional management of adult cancer patients [48]. These guidelines focus on preventing and improving malnutrition during cancer therapy, and make clinicians aware of the need to regularly screen all patients for the risk of excessive loss of weight. Particularly, there is a moderate level of evidence and a strong recommendation to support a positive protein balance with a protein intake above 1 g/kg/day and, if possible, up to 1.5 g/kg/day [48,109]. To counteract low appetite, early satiety and reduced bowel motility, ESPEN suggests increasing the energy density of the diet, replacing glucose with lipids in parenteral nutrition regimens. Reducing carbohydrates seems to be beneficial for cancer patients to limit the infectious risk related to hyperglycemia [48]. *N*-3 fatty acids emulsion could be useful to this purpose, acting as an antioxidant and appearing less proinflammatory than other *N*-6-based fatty emulsions [48,110]. However, the lack of clinical studies comparing the effects of different fat emulsions does not allow to formulate a strong recommendation. Finally, the risk of micronutrient deficiency is within all forms of malnutrition and cancer patients should refer to recommendations of international societies, such as the World Health Organization, for daily intake of micronutrients [111,112].

## 6. Role of Diet in Secondary Prevention of Cardiovascular Disease in Cancer Survivors: A Few Stones Left Unturned

Early detection of cardiac damage, proper early treatment and long-term follow-up are key strategies in the secondary prevention of the progressive cardiovascular alteration induced by antineoplastic drugs [113]. Indeed, an adequate screening combined with a prompt therapeutic management is a cost-effective strategy that leads to a reduction in the cumulative incidence of heart failure in childhood cancer survivors, and improves life quality and expectancy [104].

Cardiovascular monitoring strategies have been defined in adults exposed to cancer drugs associated with a high risk of cardiotoxicity, including anthracyclines, human epidermal growth factor-2 inhibitors, vascular endothelial growth factor inhibitors, Bcr-Abl kinase inhibitors, proteasome inhibitors, immune checkpoint inhibitors and ibrutinib. Cardio-oncological evaluation, defined as a global and standardized cardiovascular assessment strategy, includes risk factor assessment, ECG, biomarkers, and imaging evaluation [114,115]. A comprehensive assessment of modifiable cardiovascular risk factors, such as obesity, smoking, hypertension, diabetes and dyslipidemia is crucial [114]. In childhood cancer survivors, strategies for secondary prevention of cardiotoxicity have been developed in several practice guidelines for cardiotoxicity risk assessment, timing and modality for cardiovascular screening and therapeutical management [116,117,118,119,120].

Among the procedures recommended for an early detection of cardiac damage in childhood cancer survivors, echocardiography should be primarily considered and performed at baseline, no later than 2 years after exposure, and at minimum every 5 years thereafter [116]. Furthermore, the detection of asymptomatic cardiac alteration and early myocardial dysfunction can be significantly improved through the implementation of global longitudinal strain [8,121,122]. Other useful tools for the evaluation and monitoring of cardiovascular damage induced by antineoplastic drugs include cardiac magnetic resonance [115,123] and a rational use of cardiac biomarkers (i.e., troponin I, B-type natriuretic peptide and *N*-terminal prohormone of brain natriuretic peptide) [113,124].

General recommendations gathered by international guidelines [116,117,118,119,120] recommend a regular screening for modifiable cardiovascular factors, including overweight, diabetes, dyslipidemia and hypertension in this fragile population [116,125,126,127,128].

In this perspective, age-specific and customized nutritional interventions on childhood cancer survivors should be deeply encouraged [127].

With regard to specific nutritional interventions for the secondary prevention of cardiovascular damage in childhood cancer survivors, there is a lack of studies investigating specific dietary compounds and detailed interventions in this fragile population. Furthermore, only a few heterogeneous studies have been published on nutritional interventions in childhood cancer survivors [127], forcing pediatricians to rely on adult studies for guidance. General recommendations could be gathered to that formulated by the American Cancer Society [129,130] that offers practical advice for the purpose of a healthy lifestyle. Particularly, limiting high-calories foods and beverages should be suggested in overweight or obese patients. Daily intake of healthy foods from plant sources should be encouraged as well as a regular consumption of vegetables and fruits (circa 300 g, at least five portions of fruit and vegetables per day) [116] and a limited ingestion of processed meat and red meat [129]. In adolescence and adulthood, high alcohol intake and smoking should be strongly discouraged [116,131,132] and the benefits of a regular physical activity should be stressed. Indeed, it is recommended to return to normal daily activities as soon as possible after diagnosis, exercising at least 150 min per week, and including strength training exercises at least 2 days per week [129].

Specific nutritional programming for high-risk populations, based on digitally delivered cooking interventions such as online cookbooks, could represent a useful tool in helping cancer survivors and their families adopt a healthier diet [133].

Interestingly, bad childhood dietary habits extend to the working-age adults (age 18–64), in which there is a higher prevalence of food insecurity, the inability to access nutritionally and culturally adequate food with subsequent unbalanced meals that are frequently a consequence of financial difficulties. Since socio-demographic factors are strongly related to food insecurity, the development of food assistance programs and networks could have a great impact on these patients’ health [127].

In conclusion, nutritional and life-style recommendation aimed to reduce cardiovascular risk in childhood cancer survivors can be related to that of general population [134].

To our knowledge, there is a lack of studies focusing on nutritional interventions for secondary prevention of cardiovascular disease in childhood cancer survivors. Future research includes the identification of potential nutritional interventions or specific dietary compounds with a cardioprotective effect on childhood cancer survivors, especially those with drug-related cardiotoxicity.

## 7. Conclusions

Childhood cancer survivors are a growing population that will demand social, clinical and scientific attention in the future. Due to their long-life expectancy and their high sensitivity to antineoplastic toxicity, children who survived cancer are at higher risk for cardiovascular complications and a poor quality of life.

Nutrition and dietary intervention are already recognized as key factors in secondary prevention and clinicians are aware of the cruciality of a customized strategy to ameliorate cancer survivors’ lifestyle. Nevertheless, evidence of a possible role of dietary products in primary prevention are arising, opening new perspectives in cardiotoxicity induced by cytotoxic agents. The chance to reduce cardiotoxicity through nutrition is merely theoretical but undoubtedly fascinating. Further studies and clinical evidence are needed to prove the efficacy of the citated potential cardioprotective dietary products.

## Figures and Tables

**Table 1:** List of nutrients with a potential role in primary prevention of cardiovascular damage in childhood cancer survivors, with their sources, properties and additional protective effects.

		Sources	Properties	Protective Effects
**ω-3 PUFA**	*Podpeskar A.* et al. [54]	Marine fish (e.g., herrings, halibuts, mackerels and salmons) Vegetable oils Seeds (e.g., flax, canola and soy)	Antioxidant Anti-inflammatory Anti-angiogenetic Antiproliferative Proapoptotic	Prevention of lipid dysregulation, alteration in glucose metabolism and low-grade inflammation related to the long-term disease following cytotoxic therapies (potential prevention of ACT) Maintenance of gut health Cancer growth inhibition
**CoQ_10_**	*Conklin K.A.* [65] *Zahra K.F.* et al. [66] *Iarussi D.* et al. [67]	Oily fish (e.g., salmon and tuna) Organ meats (e.g., liver) Whole grains	Antioxidant Antiproliferative	Cardioprotective effect Prevention of ACT Cancer growth inhibition
**EGCG**	*Dudka* et al. [68] *He H.* et al. [69]	Green tea Other teas (white, oolong, and black). Some fruits (e.g., cranberries, strawberries, blackberries, kiwis, cherries, pears, peaches, apples and avocados)	Antioxidant Anti-angiogenic Antiproliferative Proapoptotic Cell motility inhibition	Prevention of ACT Reduction of intracellular damage due to antineoplastic therapies Cancer growth inhibition
**Quercetin**	*Psotová J.* et al. [70] *Du G.* et al. [71] *Tang S.M.* et al. [72] *Psotová J.* et al. [73] *Václavíková R.* et al. [74]	Some fruits (e.g., citric fruits, berries, grapes, cherries) Tea Olive oil Onions Vegetables (e.g., broccoli)	Antioxidant Anti-inflammatory Antiproliferative Proapoptotic Anti-angiogenic	Cardioprotective effects Prevention of ACT Cancer growth inhibition Other cytoprotective effects on liver and spleen
**Genistein**	*Bai Z.* et al. [75] *Chen M.* et al. [76]	Soy-based foods (e.g., soy cheese and soy drinks)	Antioxidant Antiproliferative Anti-inflammatory Anti-carcinogenic Anti-adipogenic Regulator of insulin sensitivity, fatty acid metabolism and adipocyte differentiation	Prevention of ACT Chemopreventive effects Downregulation of type II diabetes, artherogenesis and obesity
**Curcumin**	*Venkatesan N.* et al. [77] *Swamy A.V.* et al. [78] *Wang N.P.* et al. [79] *Farhangkhoee H.* et al. [80] *Motterlini R.* et al. [81]	Curry spice turmeric	Antioxidant Anti-inflammatory Anti-thrombotic Antiproliferative Anti-carcinogenic	Prevention of ACT Prevention of diabetic complications Stabilization of cardiac cell membrane Maintenance of cholesterol homeostasis Chemopreventive effects Cardioprotective effects
**Resveratrol**	*Maayah Z.H.* et al. [82] *Cao Z.* et al. [83] *Zhang L.* [84] *Gu J.* et al. [85] *Matsumura N.* et al. [86]	Grapes Red wine Cocoa and chocolate Peanuts Some berries (e.g., blueberries, bilberries and cranberries)	Antioxidant Antiproliferative Anti-inflammatory Anti-angiogenic	Prevention of ACT Cardioprotective effects Cancer growth inhibition
**L-carnitine**	*Mansour H.H.* et al. [43] *Bayrak S.* et al. [87] *Aziz M.M.* et al. [88]	Red meat, poultry Fish Dairy products	Antioxidant Anti-inflammatory	Prevention of ACT Cardioprotective effects Protective role against drug-induced neurotoxicity, nephrotoxicity and ototoxicity
**Glutathione**	*Mohamed H.E.* et al. [89]	Cruciferous vegetables (e.g., brussels sprouts, broccoli, cauliflower, kale, watercress, mustard greens and spinach) Other vegetables (e.g., asparagus, okra) Avocados Shallots, garlic, onions Poultry, beef Fish	Antioxidant	Potential role in preventing ACT
**Vitamin A**	*Khafaga A.F.* et al. [90]	Dairy products (milk, yoghurt, cheese) Eggs Oily fish Liver *Sources of provitamin A, that the body can convert into vitamin A:* Yellow, red and green vegetables (e.g., spinach, carrots, sweet potatoes and red peppers) Yellow fruits (e.g., mango, papaya and apricots)	Antioxidant Anti-inflammatory Antiproliferative Pro-differentiation Pro-apoptotic	Prevention of ACT Chemopreventive effects Cancer growth inhibition
**Lycopene**	*Yilmaz S.* et al. [91] *Karimi G.* et al. [92] *Ateşşahin A.* et al. [93]	Tomatoes and tomato products Some fruits (watermelon, pink grapefruit, pink guava, papaya, mangos, persimmon, dried apricots, pureed rosehips) Sweet red peppers Red cabbage Asparagus	Antioxidant Anti-inflammatory	Cytoprotective effect on heart, testicular and kidney tissues Potential protective effect against diabetes and obesity
**Vitamin E**	*Wartenberg* et al. [94] *Conklin* et al. [11] *Puri A.* et al. [95]	Plant-based oils (e.g., wheat germ oil, safflower oil, and sunflower oil) Seeds (e.g., sunflower seeds, almonds) Dry Fruits (e.g., nuts, peanuts) Other fruits (e.g., mango, avocado, and pumpkin) Vegetables (e.g., beet greens, collard greens, spinach, asparagus and red bell peppers)	Antioxidant Anti-inflammatory Antiproliferative Pro-apoptotic	Potential role in preventing ACT Chemopreventive effects
**Vitamin C**	*Wouters* et al. [12] *Akolkar* et al. [96] *Van Dalen* et al. [97]	Citrus fruits (e.g., oranges, kiwi, lemon, grapefruit) Other fruits (e.g., strawberries) Cruciferous vegetables (e.g., broccoli, brussels sprouts, cabbage, cauliflower) Other vegetables (e.g., bell peppers, white potatoes, tomatoes)	Antioxidant	Protective role against chemotherapy-associated toxicity Cancer growth inhibition

Legend: ω-3 PUFA stands for omega-3 polyunsaturated fatty acids; ACT stands for anthracycline-related cardiotoxicity; CoQ_10_ stands for coenzyme Q_10_; EGCG stands for epigallocatechin gallate.
